# Pilot Study on the Influence of Incentive Spirometry on Percutaneous Image-Guided Intra-Abdominal Drainage Catheter Pressure: A Potential Method to Enhance Drainage

**DOI:** 10.3390/app13127308

**Published:** 2023-06-20

**Authors:** Aravinda Ganapathy, David H. Ballard, Grace L. Bishop, Mark J. Hoegger, Nihil Abraham, Horacio B. D’Agostino

**Affiliations:** 1 School of Medicine, Washington University School of Medicine, St. Louis, MO 63110, USA;; 2 Mallinckrodt Institute of Radiology, Washington University School of Medicine, St. Louis, MO 63110, USA;; 3 Department of Internal Medicine, University of California Riverside School of Medicine, Riverside, CA 92521, USA;; 4 Department of Radiology, Louisiana State University Health Shreveport, 1501 Kings Highway, Shreveport, LA 71103, USA;

**Keywords:** incentive spirometry, intra-abdominal fluid collection, percutaneous drainage

## Abstract

**Background:**

To report the evaluation of incentive spirometry (IS)-induced pressure changes in intra-abdominal drainage catheters and consider its use for maintaining catheter patency and enhancing drainage.

**Methods:**

Prospective study of patients with indwelling intra-abdominal drainage catheters for abdominal fluid collections who had their intra-abdominal pressures measured while performing incentive spirometry. Patients were instructed in the use of an incentive spirometer. Within a week after initial drainage, pressure changes with IS were evaluated three times at 1500 cc and three times at maximum inspiratory effort. Intra-abdominal pressure (IAP) was measured using a pressure monitor connected to the drainage catheter.

**Results:**

Twenty patients (men, 12; women, 8). Fluid collection locations were pelvis, Right-upper quadrant (RUQ), Left-upper quadrant (LUQ), Left-lower quadrant (LLQ), and Right-lower quadrant (RLQ). A total of 16 of 20 patients showed an elevation of IAP with IS. At 1500 cc, the pressure increased by an average of 41.24 mmH_2_O. At maximal inspiratory effort, the pressure increased by an average of 48.26 mmH_2_O. Pressure increase was greater in upper abdomen catheters. Four patients with lower abdominal and pelvic collections showed minimal pressure changes with IS.

**Conclusion:**

IS increases IAP and fluid flow through abdominal drainage catheters. Future studies are warranted to determine whether the use of IS enhances catheter performance and facilitates drainage via its effect on IAP.

## 1. Introduction

Image-guided percutaneous catheter drainage (PCD) performed by interventional radiologists is commonplace for the evacuation of most abdominal fluid collections [1–10]. This is especially true for spontaneous and postoperative abscess evacuation. A Medicare study spanning from 2001–2013 showed a substantial increase in the proportion of intra-abdominal abscesses treated by percutaneous drainage by radiologists compared to surgical drainage [11]. PCD has proven more efficient than both open surgical drainage of abscesses as well as needle aspiration [12]. When compared with needle aspiration, percutaneous catheter drainage yields an improved cure rate and reduces the abscess cavity [12,13]. Unlike open surgical drainage, percutaneous fluid drainage offers both noninvasive localization and minimally invasive collection of fluids without general anesthesia. Morbidity, mortality, and length of hospital stay are also all decreased with PCD relative to open surgical drainage [13]. PCD has a success rate of 90% and a generally low (<5%) incidence of complications, depending on the nature of the abscess [12]. Despite its success rates, the drawbacks of PCD include occlusion of catheter side holes, infections, bleeding, and stagnation of fluid flow; the latter of which can be ameliorated by increasing the intra-abdominal pressure relative to the pressure outside the body [12,14].

**Figure 1.**
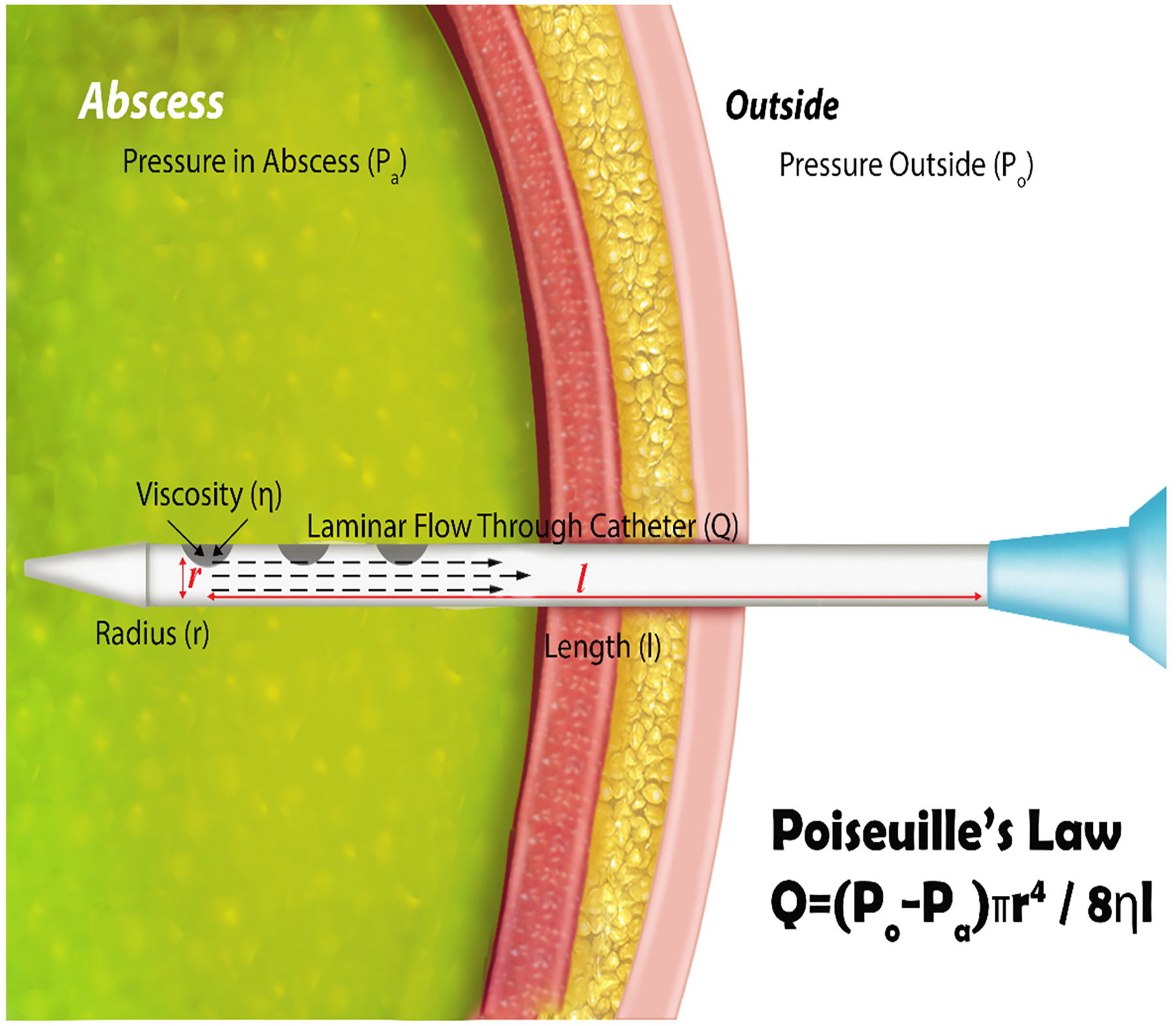
Intra-abdominal abscess drainage as depicted. An increase in intra-abdominal pressure results in an increase in *P*_1_, which we theorize should result in a *Q*_*Laminar*_ increase. Reproduced with permission from reference [1].

Fluid flows through drainage catheters from inside the body to the external collection bag in a laminar fashion according to Poiseuille’s Law $\left(Q_{\text{Laminar}}=\frac{(P_{2}-P_{1)\pi R^{4}}}{8\eta \iota }\right)$ (Figure 1). A greater pressure differential (*P*_2_ − *P*_1_, Δ*p*) and a lower length factor (*r*) increase the flow of fluid through the catheter.

Spirometry breathing exercises cause variations in the intra-abdominal pressure. Increased intra-abdominal pressure is known to augment catheter drainage [15]. The use of incentive spirometry to potentially enhance catheter drainage by increasing intraabdominal pressure has not been studied before. Our study focuses on a novel and potentially impactful use of incentive spirometry to enhance percutaneous catheter drainage. Prior research has shown that increased intra-abdominal pressure can improve catheter drainage, but no prior studies have examined the potential of IS-induced pressure changes to enhance PCD. The purpose of this study is to report the evaluation of incentive spirometry-induced pressure changes in intra-abdominal drainage catheters and consider their use for enhancing drainage; this represents an area of considerable significance since it has the potential to substantially impact clinical practice by optimizing an already-effective procedure in IS.

## 2. Materials and Methods

This was an IRB-approved, Health Insurance Portability and Accountability Act compliant, prospective observational study evaluating the intra-abdominal pressure of patients with percutaneous drainage catheters for evacuation of intra-abdominal fluid collections while actively performing incentive spirometry. Eligible patients included those with clinical indications for image-guided percutaneous drainage, admitted to the hospital while having daily evaluations of their percutaneous drains, and those who met the minimum inspiratory capacity equal to or greater than 1500 mL (described subsequently). Informed consent to participate in this study was obtained from all patients.

**Figure 2.**
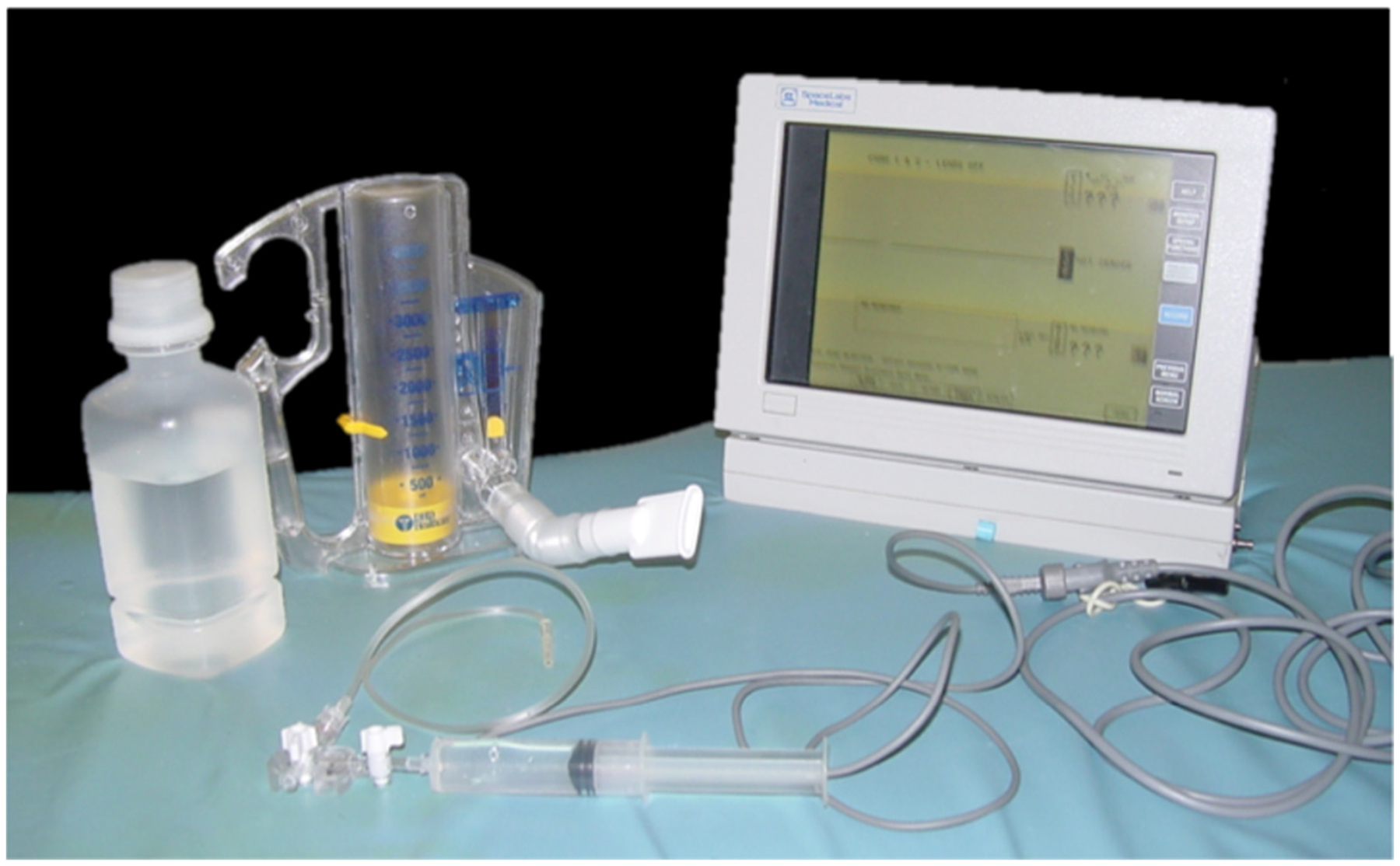
Equipment Used in Measurement of IAP via Incentive Spirometry. From left to right: saline, incentive spirometer, pressure readout monitor, stopcock connection to drainage catheter (shown in front).

Twenty patients (men, 12; women, 8; age range, 23–69 years; average 46 years) with intra-abdominal drainage catheters enrolled in this study. Catheters were inserted in the abdominal cavity for fluid collection and evacuation (*n* = 20). Fluid collections drained were: tubo-ovarian abscesses (*n* = 5); postoperative pelvic abscesses (*n* = 2); ascites (*n* = 2); pancreatic abscesses (*n* = 2); bilomas (*n* = 1); pancreatic pseudocysts (*n* = 2); hepatic hematoma (*n* = 1); diverticular abscess (*n* = 1); pelvic cyst (*n* = 1); liver abscess (*n* = 1); subphrenic abscess (*n* = 1). Collections were located in the pelvis (*n* = 7); RUQ (*n* = 4); LUQ (*n* = 4); LLQ (*n* = 3); RLQ (*n* = 1). Figure 2 demonstrates the insertion of the drainage catheter for possible abscesses in the abdominal and pelvic regions (Figure 2). There were 2 patients who did not qualify for study inclusion as they were unable to meet the minimum tidal volume inspiratory capacity equal to or greater than 1500 mL; thus, intra-abdominal pressure was measured in 18 patients.

Patients were instructed in the use of an incentive spirometer by respiratory therapists and spirometer-drainage catheter experiments were performed under the supervision of an attending board-certified interventional radiologist and a radiology resident. Within a week after drainage, intra-abdominal pressure changes were evaluated by catheter 3 times after a 1500 mL inspiratory pull with incentive spirometry, and also measured 3 times after maximum inspiratory effort. Participants were first told to inspire until the spirometer read 1500 mL, and then told to inspire to their maximum capacity. Criteria selection for patients to enter the study was an inspiratory capacity equal to or greater than 1500 mL. Intra-abdominal pressure was recorded using a pressure monitor connected to the drainage catheter. Changes in intra-abdominal pressure through the catheters with incentive spirometry were measured and compared with baseline tidal volume (0 mmH_2_O). The equipment used is shown in Figure 2.

**Figure 3.**
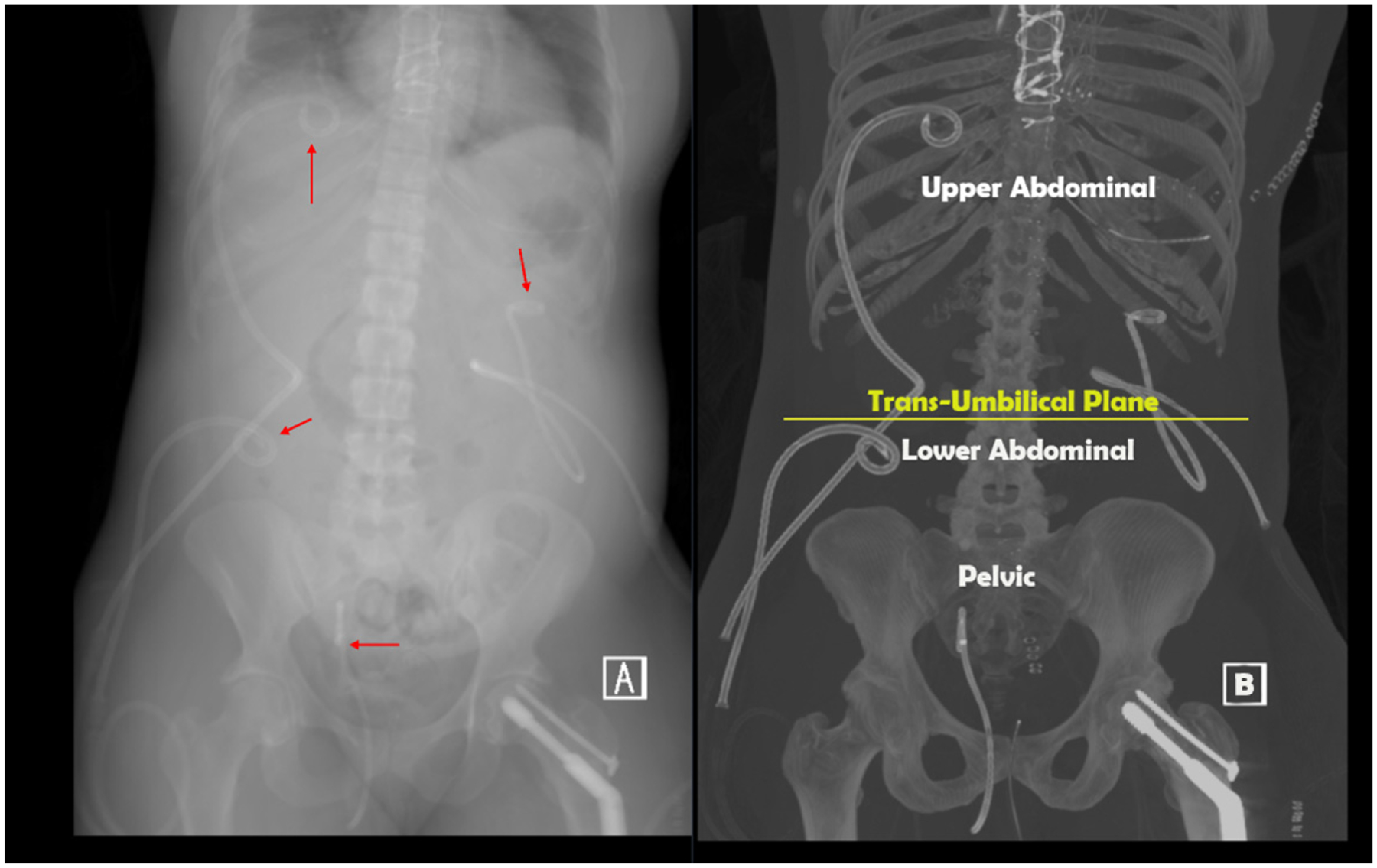
(**A**,**B**). Upper and lower abdominal drainage catheters as defined by the study criteria. A 30-year-old woman was admitted with blunt polytrauma following a motor vehicle collision with multiple injuries complicated by a long intensive care unit admission and multiple infected abdominal and pelvic fluid collections related to bowel and pancreatic injuries evacuated with drainage catheters. (**A**) Red arrows denote the location of 4 drainage catheters at variable distance from the diaphragm: 3 percutaneous abdominal catheters, 2 in the upper abdomen above the trans-umbilical plane, and 1 in the lower abdomen below the plane; a single transrectal drainage is in the lower pelvis.

Drainage regions were defined as either upper abdominal or lower abdominal based on whether they were above or below the transumbilical plane (an anatomical plane that passes through the umbilicus), or pelvic if they were placed transrectally within the pelvic region. Epigastric drainage was not conducted in this study. A case study below demonstrates the regions as defined in Figure 3.

The results of the study were grouped by region, comparing pressure increases within the standardized inspiration group and then within the max inspiration group. Statistical analysis was conducted using R Studio version 1.3.1056, and graphs were made using GraphPad Prism version 8.0.0 for Windows, GraphPad Software, San Diego, CA, USA, www.graphpad.com, accessed on 8 October 2022. Non-parametric analyses were used due to the low sample size in each group. A one-way ANOVA with post-hoc Dunn’s non-parametric analysis was performed to determine the significance between intra-abdominal pressure in drainage catheter regions for standard inspiratory effort. Multiple linear regression was used to model IAP during maximum inspiration in different catheter drainage regions, and ANCOVA was used to compare averages.

## 3. Results

Elevation of the intraabdominal pressure with ICS was demonstrated in 16 of 18 patients.

**Figure 4.**
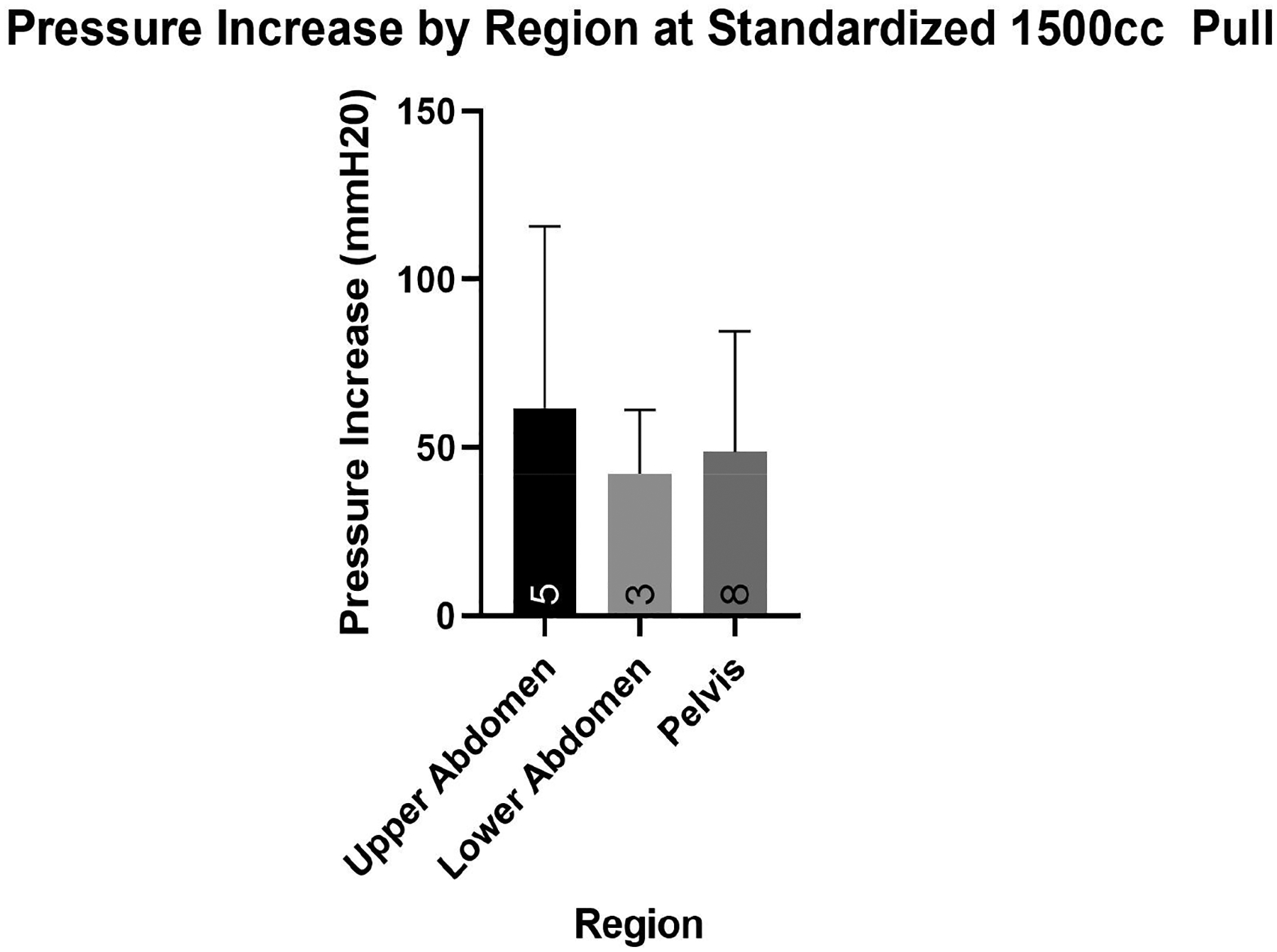
Upper abdomen catheterization non-significantly greater than lower abdomen and pelvic catheterization (*p* > 0.05). Sample sizes are listed at the bottom of each bar.

The region of catheterization was divided as either upper abdominal (*n* = 5, 25%), lower abdominal (*n* = 3, 15%), or pelvic (*n* = 8, 40%). At a standardized pull of 1500 cc, there was no significant difference in pressure increase by region (*p* > 0.999 in all comparisons, Figure 4).

**Figure 5.**
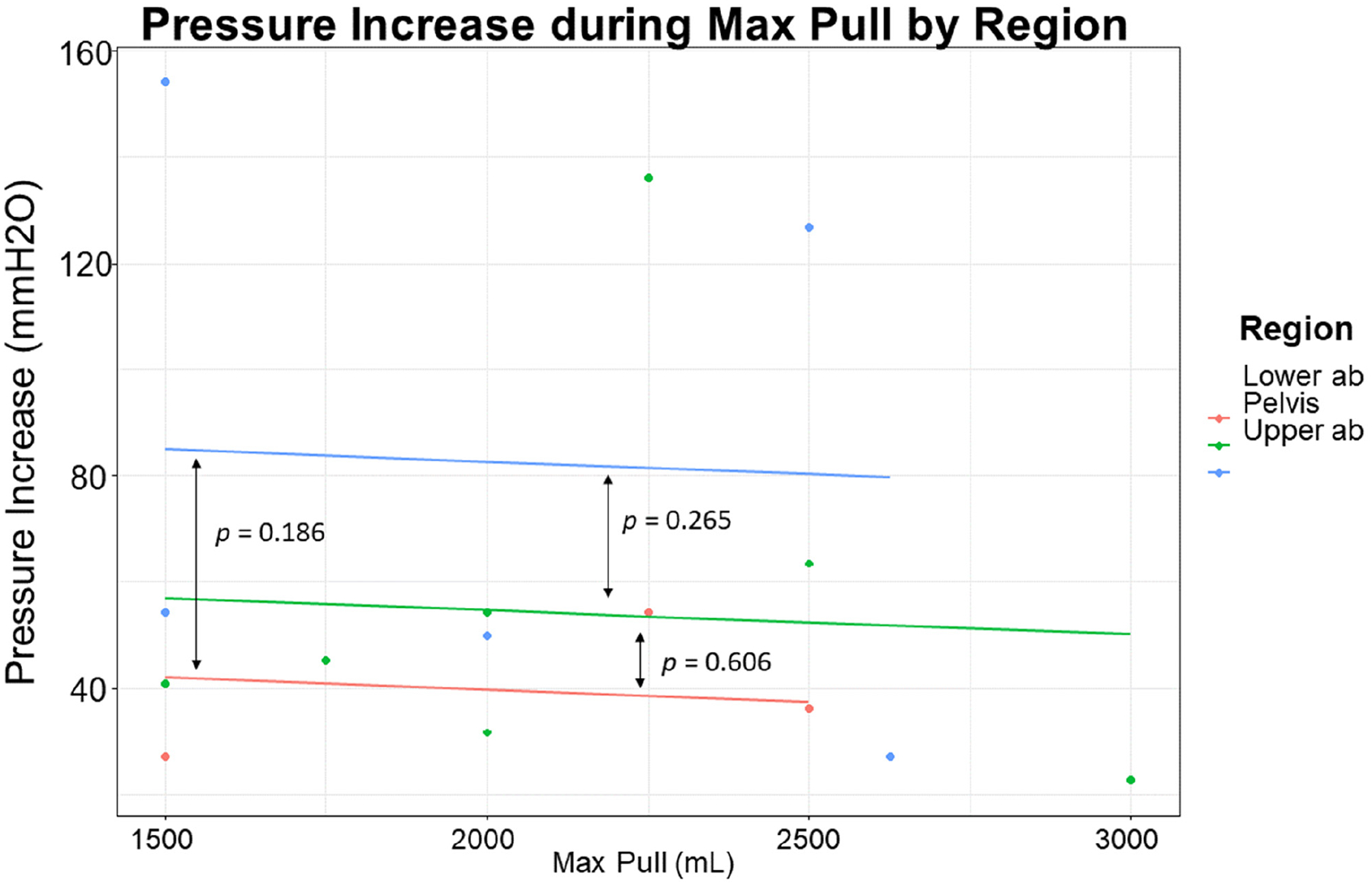
Upper Abdomen catheterization non-significantly greater intra-abdominal pressure increase than lower abdomen or pelvic catheterization. *p* values between regions are listed between lines.

ANCOVA regression is graphically depicted comparing the pressure increases by region in Figure 5; *p* values are demonstrated between lines. At maximal inspiratory effort (average pull 1999 cc), pressure increase was non-significantly (*p* value range = 0.186 to 0.606) greater in upper abdomen catheterization than in lower abdominal or pelvic catheterization (adjusted R-squared = −0.044).

Overall, pressure differences were not significantly different between regions of catheterization for standardized pull or maximal inspiratory effort. Average pressures ± standard deviation for standardized pull and max pull are shown in Table 1. The highest intra-abdominal pressure changes were seen in the upper abdomen (61.6 for standardized pull and 82.5 for max pull) and the lowest intra-abdominal pressure changes were seen in the lower abdomen (42.3 for standardized pull and 39.3 for max pull). However, none of the regional changes in intra-abdominal pressure were significantly different from each other.

ANCOVA regression was again used to determine any contribution of differential fluid collections to pressure changes during maximal inspiratory effort. There were no significant differences in intra-abdominal pressure changes during maximal inspiratory effort between any of the fluid collections from each other (*p* > 0.05). Mean pressure differences by fluid collection are shown in Table 2.

## 4. Discussion

Our study shows an increase in abdominal pressure with the use of IS. This effect was non-significantly more accentuated in the upper abdomen than the lower abdomen or pelvis. Therefore, it was found that depending on the location and type of the fluid collection, drainage catheter performance may be enhanced by maintaining catheter patency and by the elevation of the intra-abdominal pressure while using IS for additional circulatory and respiratory benefits (see below). In contrast to previous studies, our study is the first to examine and compare differential elevations in drainage pressure between different abdominal and pelvic regions [16,17]. This information is important for determining the potential locations and efficacy of intra-abdominal drainage and demonstrates the potential utility of incentive spirometry in facilitating intra-abdominal drainage. Furthermore, while incentive spirometry, intra-abdominal pressure, and drainage catheters have each been subjects of individual study, the unique aspect of the current research is the exploration of the interconnected relationship between all three components.

Relative to atmospheric pressure, increased intra-abdominal pressure promotes fluid flow from the body to the collection bag. After substantial drainage has occurred, the intra-abdominal pressure and the pressure outside the body will be in equilibrium. At this point, the fluid in the lumen of the catheter will be stagnant; Further increasing the intra-abdominal pressure fosters fluid flow from within the abdomen to the collection bag, increasingly draining the collection.

It is important to consider the inspiratory effort in the context of the different fluid collections. One study demonstrated differential viscosities within different fluid collections, with increasing viscosities as follows: water, pseudocysts, blood, and abscess fluid as the most viscous [18]. However, we did not find any significant difference between various fluid collections despite their different viscosities. It is also important to consider that fluid collection viscosities can vary greatly depending on the individual and their body type and pathology; since we did not explicitly measure the fluid viscosities or densities, we can only draw a comparison between the broad categories and cannot specifically control for the densities.

An additional benefit of incentive spirometry (IS) is the abdominal postoperative prevention of respiratory complications and venous stasis. Upper abdominal surgeries are associated with a functional residual capacity (FRC) reduction of approximately 35%, while lower abdominal surgeries are associated with a 10–15% FRC reduction [19,20]. Pain from the abdominal wound and impaired respiratory muscle function may persist in these patients for up to a week before gradually returning to baseline. The reduced activity of these muscles is more frequently observed in upper abdominal surgeries compared to lower (20–40% vs. 2–5%, respectively) [16,21,22].

The most common complications following upper abdominal surgery are pulmonary complications (such as atelectasis) [16,20,23,24], with incidence ranging as high as 80% [16,24,25]. To treat or prevent these complications, pulmonary physical therapy is often prescribed [26–30]. Common pulmonary physical therapy methods used to treat or prevent these complications include incentive spirometry (IS), deep breathing exercises (DBEX), and intermittent positive pressure breathing (IPPB). IS improves lung aeration and expansion, leading to protection against respiratory complications. Furthermore, post-operative venous stasis can be alleviated by deep inspiration, which increases the venous blood return to the heart [31].

According to Hodges and Gandevia and a study conducted by Takimoto et al. [32], the movement of the diaphragm in conjunction with the contraction of abdominal and pelvic floor muscles causes an increase in intra-abdominal pressure during inspiration [32–34]. The prolonged inspiration of incentive spirometry leads to a subsequent increase in abdominal pressure. An elevation in abdominal pressure can assist with drainage catheter patency, which presumably assists in the full evacuation of fluid collections. It has been shown that intra-abdominal pressure increases after abdominal surgery [16,35,36] and also during inspiration [33,34] and other physical movements such as ambulation [28,37]. The physics and fluid mechanics of drainage catheters have been previously described as following Poiseuille’s law [14]. Previous studies describe the effect that decreased length and increased catheter diameter can have on flow [14,38]. As a result, it may be prudent to not only consider the use of incentive spirometry to increase the pressure differential, but also in conjunction with the use of a short tube. Our results align with these findings.

There are several limitations to the present study. Most notably, the flow rate through the drainage catheters was not measured, neither during baseline nor during either of the incentive spirometry therapies. Secondly, this study only describes a patient population with a single drainage catheter; no data was gathered for patients with multiple catheters. Finally, the study is constrained by a small sample size due to resource limitations and enrollment criteria, potentially limiting the statistical power to detect significant differences between regions. However, this study serves as an important initial step in raising pertinent questions regarding the role of incentive spirometry in managing intra-abdominal pressure. The findings provide a rationale for future larger-scale studies. Additionally, the data is derived from a single institution, as the study was originally designed as a feasibility investigation to evaluate the impact of incentive spirometry on intra-abdominal pressure within our institution. Limited resources, such as time and funding, hindered our ability to coordinate a multi-center study at this stage. However, we believe that our findings provide a valuable foundation for future multi-center studies that can further explore and validate our results. Incentive spirometry is prescribed differently by each physician, and results from additional patients could vary depending on the abscess type as well as individual patient compliance.

Finally, it is important to note a methodological constraint pertaining to the classification of intra-abdominal drainages. The present study demarcates upper and lower drainages based on their positioning in relation to a horizontal plane passing through the umbilicus. The variability in individual anatomical configurations, particularly with mobile structures such as the bowel attached to the mesentery, may pose a potential limitation. It is worth noting that the alignment of this line may not necessarily coincide with the projection of the transverse mesocolon, which has been proposed by some as a means to delineate the peritoneal cavity into upper and lower sections [39]. This discrepancy may lead to inconsistent classification and potential bias in the interpretation of the results, as the exact anatomical location of the drainages within the peritoneal cavity may influence the effectiveness of incentive spirometry.

Future directions for this pilot study are multi-faceted. We believe it is critical to validate our results with a larger sample size, and with a multi-center study. Of course, it is important to study the direct relationship between catheter drainage overall and the administration of incentive spirometry as well. Looking forward, it may be beneficial to broaden our research scope to include multiple types of drainage catheters. In this study, we elected to use a single type to reduce variability and focus on the effects of incentive spirometry on intra-abdominal pressure. However, recognizing that different catheters may behave distinctively and could influence results in various intra-abdominal and intra-pelvic regions, future research could endeavor to investigate these potential differences and their implications.

## 5. Conclusions

In a small cohort of patients, albeit more intraabdominal pressure elevation on drainage catheters placed in the upper than in the lower abdomen and pelvis was found, such a difference was not statistically significant. Such changes, more accentuated in the upper abdomen, are likely related to the upper abdominal organs’ increased motility due to the proximity of the diaphragm. To achieve superior drainage of intra-abdominal fluid, incentive spirometry in conjunction with a short catheter should be used according to Poiseuille’s Law. Patients with drainage catheters can undergo incentive spirometry to facilitate drainage of their fluid collections while simultaneously decreasing the risk of pulmonary complications. Our study suggests prescribing IS for patients undergoing image-guided percutaneous abdominal catheter drainage to evacuate fluid collections. Changes in intraabdominal pressure caused by IS have the respiratory and circulatory benefits detailed and may provide the additional perk of keeping the catheter patent while optimizing drainage.

## Figures and Tables

**Table 1.** Intra-Abdominal Pressure Increases in Standardized and Max Pull Groups. Intra-abdominal pressure was measured via a pressure monitor that was connected to patients’ drainage catheters. Pressure was recorded in the upper abdomen, lower abdomen, and pelvis at a standardized pull of 1500 ccs and max pull, which averaged 1999 ccs.

Intra-Abdominal Pressure Increases by Region			
Standardized Pull (1500 cc)		Max Pull (Average 1999 cc)	
Upper Abdomen		Upper Abdomen	
Mean ± STD	61.6 ± 54.2	Mean ± STD	82.5 ± 54.8
Median	54.4	Median	54.4
Min-Max	18.1–154	Min-Max	27.2–154
Lower Abdomen		Lower Abdomen	
Mean ± STD	42.3 ± 18.9	Mean ± STD	39.3 ± 13.8
Median	36.2	Median	36.2
Min-Max	27.2–63.4	Min-Max	27.2–54.4
Pelvis		Pelvis	
Mean ± STD	48.7 ± 35.9	Mean ± STD	54.4 ± 35.3
Median	40.8	Median	43.1
Min-Max	27.2–136	Min-Max	22.7–136

**Table 2.** Intra-Abdominal Pressure Increases (mmHg) in Max Pull Grouped by Fluid Collections.

	Abscess	Ascites	Biloma	Cyst	Hematoma
Mean (SD)	52.7 ± 36.3	40.8	95.2 ± 83.3	71.4 ± 37.1	27.2
Median	43.1	NA	95.2	54.4	NA
Min-Max	22.7–136	NA	36.2–154	49.8–127	NA

## Data Availability

Data is available upon reasonable request to the corresponding author.
